# A novel variant in *GATM* causes idiopathic renal Fanconi syndrome and predicts progression to end‐stage kidney disease

**DOI:** 10.1111/cge.14235

**Published:** 2022-10-21

**Authors:** Eleanor G. Seaby, Steven Turner, David J. Bunyan, Fariba Seyed‐Rezai, Jonathan Essex, Rodney D. Gilbert, Sarah Ennis

**Affiliations:** ^1^ Faculty of Medicine University of Southampton Southampton UK; ^2^ Broad Institute of MIT and Harvard Cambridge Massachusetts USA; ^3^ Wessex Regional Genetics Laboratory Salisbury NHS Foundation Trust Salisbury UK; ^4^ School of Chemistry University of Southampton Southampton UK; ^5^ Southampton Children's Hospital Southampton UK

**Keywords:** end‐stage kidney disease, exome sequencing, genetics, molecular dynamics, renal Fanconi syndrome

## Abstract

Renal Fanconi syndrome (RFS) is a generalised disorder of the proximal convoluted tubule. Many genes have been associated with RFS including those that cause systemic disorders such as cystinosis, as well as isolated RFS. We discuss the case of a 10‐year‐old female who presented with leg pain and raised creatinine on a screening blood test. Her mother has RFS and required a kidney transplant in her thirties. Further investigations confirmed RFS in the daughter. Exome sequencing was performed on the affected mother, child, and unaffected father. We identified a novel variant in *GATM*; c.965G>C p.(Arg322Pro) segregating dominantly in the mother and daughter. We validated our finding with molecular dynamics simulations and demonstrated a dynamic signature that differentiates our variant and two previously identified pathogenic variants in *GATM* from wildtype. Genetic testing has uncovered a novel pathogenic variant that predicts progression to end stage kidney failure and has important implications for family planning and cascade testing. We recommend that *GATM* is screened for in children presenting with RFS, in addition to adults, particularly with kidney failure, who may have had previous negative gene testing.

## Graphical abstract

Whole exome sequencing of a mother and daughter with isolated Fanconi syndrome revealed a novel variant of *GATM*, c.965G>C p.(Arg322Pro). Molecular dynamic simulation confirmed that the abnormal protein product would tend to form multimers likely to disrupt mitochondrial function.

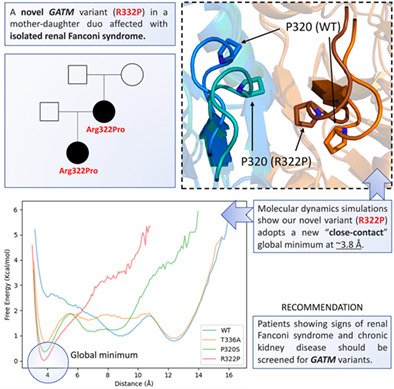

## 1 INTRODUCTION

Renal Fanconi syndrome (RFS) is a disorder of the proximal convoluted tubule, resulting in insufficient reabsorption of solutes in the proximal renal tubule, a process that is highly energy dependent. To date, several genes have been associated with RFS, of which some cause systemic disorders with RFS and others cause isolated RFS with or without kidney failure (Table S1).

The genetic aetiology of isolated RFS is still being elucidated. In 2001, Lichter‐Konecki et al.^1^ used genome‐wide linkage analysis to map the locus of autosomal dominant RFS to chromosome 15q15.3; at the time the pathogenic gene was unknown. With advances in genomic sequencing technologies, further genes have been associated with isolated RFS including *SLC34A1*,^2^ *EHHADH*,^3^ and *NDUFAF6*.^4^ In 2018, using linkage analysis and contemporary sequencing technologies, Reichold et al.^5^ identified *GATM* as the causal gene in the 15q15.3 locus. *GATM* was previously missed by Lichter‐Konecki et al. as they had erroneously excluded flanking markers which excluded *GATM*.

## 2 CLINICAL HISTORY

A 10‐year‐old white female presented with bilateral knee pain. Screening blood tests showed a raised plasma creatinine with an eGFR of 65 ml/min/1.73 m^2^. She was referred to Southampton's paediatric nephrology service. Her mother presented at a similar age with leg pain, polyuria and polydipsia. Aged 14, she was found to have rickets and was referred for further investigation. These confirmed RFS with a plasma creatinine concentration of 53 μmol/L. Aged 17 her plasma creatinine concentration had risen to 104 μmol/L and her GFR measured by EDTA plasma disappearance was 57 ml/min/1.73 m^2^. She progressed to end‐stage kidney disease and had a live, unrelated kidney transplant in 2020 (aged 38). Neither of the maternal grandparents had any features to suggest RFS and neither developed chronic kidney disease. A maternal uncle had completely normal kidney function.

Biochemical investigations of the presenting daughter revealed low plasma concentrations of bicarbonate (13 mmol/L), potassium (3.3 mmol/L), and inorganic phosphate (0.89 mmol/L). Urinary protein/creatinine ratio was elevated (124 mg/mmol) and her lactate was raised at 2.5 mmol/L. Alkaline phosphatase was also raised (540 U/L). Plasma glucose, sodium, magnesium, and parathyroid hormone were within normal ranges. She had heavy generalised aminoaciduria and raised urinary retinol binding protein/creatinine ratio (3850 μg/mmol; normal range 3.9–32 μg/mmol). She had intermittent glucosuria. A lower limb X‐ray revealed widening of the distal femoral and proximal tibial growth plates suggestive of rickets. Upper limb X‐rays were unremarkable. Abdominal and kidney ultrasound scan revealed bilateral medullary nephrocalcinosis. There were no other features to suggest a mitochondrial disorder. Normal white blood cell cystine (0.05 nmol ½cystine per mg protein) excluded cystinosis. Fructose intolerance was excluded due to absence of jaundice or vomiting. Galactosaemia was ruled out due to absence of cataracts and hepatosplenomegaly. Absence of persistent glycosuria, hypoglycaemia, and hepatomegaly excluded Fanconi–Bickel syndrome. Therefore, the patient was diagnosed with RFS of unknown aetiology. However, with her mother's progression to end‐stage kidney disease and evidence of an impaired eGFR, there was high suspicion for an autosomal dominant genetic cause with a similar disease trajectory.

Trio exome sequencing (affected mother, daughter, and unaffected father) was performed to investigate a molecular cause. Library capture was performed using Agilent SureSelect Human All Exon V6 and DNA were sequenced by Novogene. Data were processed from *fastq* to *vcf* using an automated joint‐calling pipeline, aligned to GRCh38. Quality control ensured only high‐quality variants remained for downstream analysis. Variants were annotated with Ensembl VEP v103 and the resultant joint‐called *vcf* was uploaded to a local installation of seqr (https://github.com/broadinstitute/seqr) for data visualisation, analysis, filtering, and reporting.

To filter our data, we applied the Renal Tubulopathies V 2.30 PanelApp gene panel and restricted our analysis to: a dominant inheritance pattern; all exonic variants excluding synonymous; allele frequency <0.001 from gnomAD v2.1.1^6^; and variants that passed variant quality score recalibration.

A total of 96 894, 96 370, and 96 830 variants were called in the daughter, mother, and unaffected father respectively. Average read depth was 53, 49, and 49 respectively. Post‐filtering, two variants remained (Table 1).

**TABLE 1:** Results of primary analysis

Chr	Position	Gene	Variant	gnomAD(g)	TOPMED	CADD	ClinVar	Proband	Mother	Father
11	128 839 289	*KCNJ1*	NM_153766.3: c.955C>T p.(Arg319Ter)	7.96E‐05	3.98E‐05	37	Likely pathogenic	Het	Het	WT
15	45 366 059	*GATM*	NM_001482.3: c.965G>C p.(Arg322Pro)	0	0	32	N/A	Het	Het	WT

Abbreviations: chr, chromosome; gnomAD(g), gnomAD genomes frequency; gnomAD, gnomAD v2.1.1 exomes frequency; Het, heterozygous; pos, position; WT, wild type.

The variant in *KCNJ1* was considered an incidental finding and not causal for RFS. Pathogenic variants in *KCNJ1* are autosomal recessive^7^ and both affected individuals are heterozygous. The novel missense variant in *GATM*: c.965G>C p.(Arg322Pro), had a genotype quality of 99 in all sequenced individuals and a depth of 74 in the proband, 74 in the mother, and 37 in the father. The variant was confirmed in the proband and mother by Sanger sequencing.

### 2.1 Validation of p.(Arg332Pro)

**FIGURE 1:**
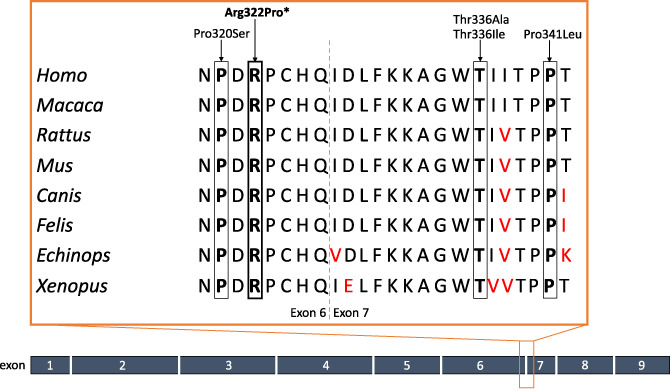
Molecular dynamics results of GATM B4‐B4 dimer mutants. (A) Graphical representation of GATM wild‐type (WT) homodimer, each monomer coloured blue and orange, dimerized at the B4–B4 interface. (B) Representative cartoon structures of 316–324 loop for WT (blue/orange) and R322P (purple/brown) global minima with respect to residue 320 distances, proline 320 residues are overlaid as a stick representation. (C) Free‐energy values for all mutants and WT across residue 320 CB atom distances, averaged across all three repeats. (D, E) Free‐energy values for WT and p.(Arg322Pro) for each simulation replica for WT and p.(Arg322Pro) simulations across residue 320 CB atom distances [Colour figure can be viewed at wileyonlinelibrary.com]

To validate the pathogenic effect of the p.(Arg322Pro) variant, we performed conventional molecular dynamics simulations on wildtype (WT), p.(Arg322Pro) (our variant), and two pathogenic variants, p.(Thr336Ala) and p.(Pro320Ser), as reported by Reichold et al.^5^ Simulations were tested on homodimers, dimerized at the multimeric B4–B4 interface as per Reichold et al.^5^ (Figure 1A) and run for 600 ns per replica, with three replicas per *GATM* variant. The classical dimer B2–B2 interaction also required for multimerization was not included in simulations as we expect this interaction to be invariant across both pathogenic and non‐pathogenic states due to its existence in the native form, and our prior simulations of B2–B2 dimers did not indicate any notable differences between variants. Further simulation details are provided in the Supporting Information. Initial principal component analysis showed no discrepancy between *GATM* variants on large scale motions observed in the trajectories (Figure S1). On visualisation of the local environment around the variant sites, p.(Arg322Pro) simulation trajectories identified unique close contacts between residues 320 on each monomer of the homodimer relative to WT (Figure 1B). Relative free energy estimations were performed across the residue 320 distance as described in the Supporting Information for all variants (Figures 1C and S1).

## 3 DISCUSSION

*GATM* encodes a proximal tubular nuclear‐encoded mitochondrial enzyme, glycine amidinotransferase, which catalyses the transfer of a guanidino group from l‐arginine to glycine, resulting in guanidinoacetic acid, an immediate precursor to creatine.^8^ GATM is most prominently expressed in kidney, liver, pancreas, and brain. In the kidney, *GATM* expression is limited to the highly energy dependent proximal tubular cells, which have abundant mitochondria to support the oxygen‐dependent generation of ATP.^5^ Biallelic loss‐of‐function variants in *GATM* cause a rare, congenital neurological disorder without kidney dysfunction.^9^

In 2018, Reichold et al.^5^ used genome‐wide linkage analysis and sequencing studies on 28 patients from five extended families with childhood‐onset autosomal dominant without debilitating rickets. The youngest patient exhibited laboratory features of RFS without glomerular compromise at 18 months old. For all patients, plasma creatinine started to rise during late adolescence or adulthood, with evidence of renal fibrosis and kidney disease. Progression to dialysis or transplant happened in the third to sixth decades. There were no extra‐renal features. All affected patients had a single heterozygous missense variant in *GATM* affecting highly conserved residues, segregating in an autosomal dominant pattern. The four causal variants, p.(Pro320Ser); p.(Thr336Ala); p.(Thr336Ile); and p.(Pro341Leu), were fully penetrant and clustered on conserved proline and threonine residues representing <5% of the protein. In silico modelling suggested the variants could adversely affect protein folding and cause GATM to form longitudinal multimers. Biopsy samples from affected patients demonstrated fibrosis and extremely large, filament‐filled mitochondria within proximal tubule cells. These were confirmed to contain GATM using immunogold staining.^5^ Overexpression of wild‐type *GATM* had no impact on mitochondria, but all mutants caused structural deformity to mitochondria consistent with the findings on patient biopsies. LLC‐PK1 cells (porcine proximal tubule cell line) transfected with the p.(Thr336Ala) variant had no effect on enzymatic activity and oxidative phosphorylation, but instead caused dimeric GATM to form multimers, resulting in fibrillary aggregation within the mitochondria consistent with the in silico modelling.^5^ These protein aggregates led to dysfunctional elongated mitochondria with increased reactive oxygen species, increased transcription of *NLRP3*, a component of the inflammasome, elevated pro‐inflammatory cytokine interleukin‐18, increased profibrotic factors and increased cell death. Furthermore, mutant *GATM* showed impaired protein degradation with an increased half‐life. Of note, mice haploinsufficient for *GATM* had no significant kidney phenotype, suggesting the four variants cause disease through a dominant negative mechanism. These mice did however display neurological phenotypes consistent with biallelic loss‐of‐function variants in *GATM*.^8^ Of interest, rats treated with oral creatine showed 27% reduced kidney *GATM* expression and 58% reduced protein levels. Therefore, exogenous creatine may suppress endogenous production of mutant GATM and reduce production of abnormal protein aggregates.^5^

**FIGURE 2:**
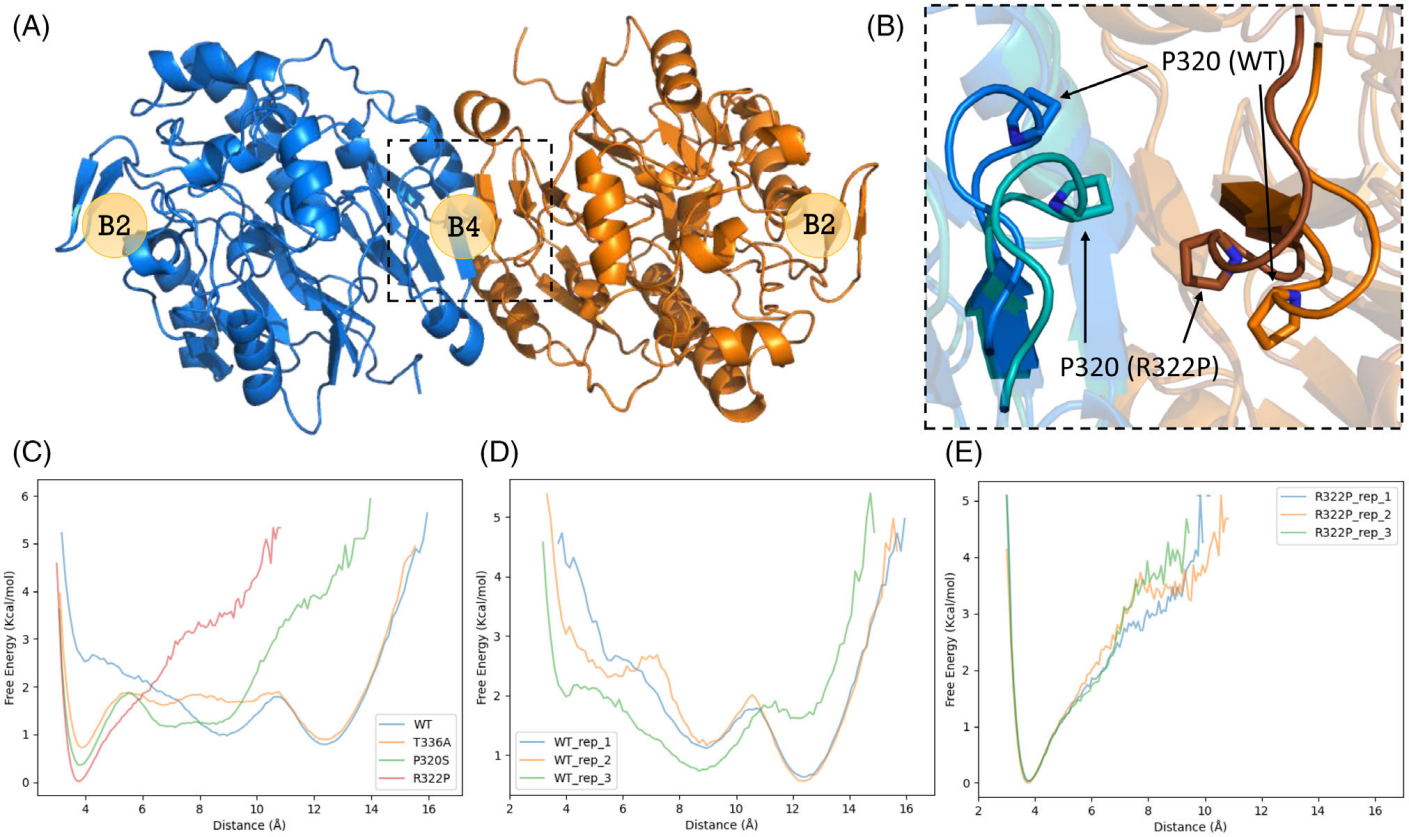
Heterozygous variants in *GATM* causing renal Fanconi syndrome with kidney failure. All known pathogenic variants in GATM, including our variant Arg322Pro (bold and with asterisk). All variants are highly conserved across species. Amino acids discordant with the human reference are shown in red. All variants span a small region of exons 6 and 7 [Colour figure can be viewed at wileyonlinelibrary.com]

We identified a novel (absent from population databases) variant, NM_001482.3: c.965G>C p.(Arg322Pro), in *GATM*, segregating in an affected mother‐daughter pair with idiopathic RFS. This variant is two amino acids downstream of a previously described pathogenic variant, p.(Pro320Ser), in *GATM* causing RFS with progression to kidney failure (Figure 2).

The p.Arg322 amino acid is extremely conserved across species, and in silico predictors suggest high pathogenicity with a CADD score of 32. To validate pathogenicity of our novel variant we performed conventional molecular dynamics simulations. We tested WT, our variant p.(Arg332Pro), and two previously identified pathogenic variants, p.(Thr336Ala) and p.(Pro320Ser). The two previously reported pathogenic variants by Reichold et al. and our novel variant behaved differently to wildtype. Disease‐associated variants adopted a new ‘close‐contact’ global minimum at ~3.8 Å, whilst WT uniquely retained a ‘distant’ global minimum at ~12.4 Å. Close contacts of residues 320 on both homodimers may be a direct or precursor event to formation of the B4 interaction surface capable of pathogenic *GATM* multimerization as hypothesised by Reichold et al.^5^ This dynamic signature localised directly at the proposed oligomeric interface, clearly stratifies disease‐associated mutants from WT in support of the pathogenicity of the p.(Arg322Pro) variant.

The phenotype of our female proband and her affected mother are consistent with the phenotypes reported by Reichold et al.^5^ The mother presented in childhood and progressed to end stage kidney disease and received a transplant in her late thirties. The daughter is already showing signs of chronic kidney disease and we expect that she will also develop end stage kidney disease and should be counselled accordingly. This has implications for family planning and the daughter may wish to undergo pre‐implantation genetic diagnosis in the future.

Currently, *GATM* (when monoallelic) is included on three gene panels in PanelApp^10^: Unexplained paediatric onset end‐stage renal disease (R257); Tubulointerstitial kidney disease (R202); and Renal tubulopathies (R198). However, it is absent from additional kidney‐related panels in PanelApp including the ‘Renal superpanel‐broad’, ‘Unexplained kidney failure in young people’ and ‘Proteinuric renal disease’. As GATM is a relatively new disease gene, we expect that there may be patients with chronic kidney disease, or end‐stage kidney failure harbouring variants in *GATM*.

## 4 CONCLUSION

All patients with known pathogenic variants in *GATM* progress to end stage kidney disease, and therefore we have uncovered a diagnosis that has serious clinical consequences for our patient and will warrant genetic counselling and cascade testing. Early functional work in rats suggests that creatine may be able to significantly reduce expression of the GATM protein and this warrants further investigation.

This case highlights how genetic sequencing and complementary molecular dynamics simulations can identify disease pathogenesis and inform patient care and prognosis. *GATM* variants should be routinely tested for in cases of idiopathic RFS even in the absence of renal failure which develops after initial presentation. We further recommend that adult patients showing signs of RFS and chronic kidney disease should also be screened for *GATM* variants.

## AUTHOR CONTRIBUTIONS

Eleanor G. Seaby processed data, performed data analysis, and wrote the first draft of the manuscript. Steven Turner, David J. Bunyan, and Jonathan Essex performed data analysis. Fariba Seyed‐Rezai assisted with data processing. Sarah Ennis, Jonathan Essex, and Rodney D. Gilbert supervised the project. All authors agreed the final manuscript.

## FUNDING INFORMATION

Eleanor G. Seaby is supported by the Gerald Kerkut Charitable Trust and the University of Southampton's Presidential Scholarship.

## CONFLICT OF INTEREST

The authors declare no conflicts of interest.

### PEER REVIEW

The peer review history for this article is available at https://publons.com/publon/10.1111/cge.14235.

## ETHICS STATEMENT

All participants were recruited to the ‘Use of NGS technologies for resolving clinical phenotypes’ study. This study was approved by Leeds East Research Ethics Committee (REC: 17/YH/0069; Protocol Number: RHM NEU0302; IRAS project ID: 212945) on the 17/05/17. Written consent was obtained from the parents of the index patient for genomic testing and from the mother for publication after she had seen the manuscript.

## Supporting information


**APPENDIX S1.** Supporting Information

**FIGURE S1.** Molecular dynamics results of GATM B4‐B4 dimer mutants
Free‐energy values for all T336A (a), P320S (b), and R322P_P322R (c) mutants across residue 320 CB atom distances, each replica plotted separately. (def) Free‐energy values for all mutants and WT across top three principal components generated from backbone Cα atom trajectories, averaged across all three replicas

**TABLE S1.** Genetic causes of renal Fanconi syndrome

## Data Availability

The data that support the findings of this study are openly available in ClinVar at https://www.ncbi.nlm.nih.gov/clinvar/.
