# Supplementary material for: A novel variant in GATM causes idiopathic renal Fanconi syndrome and predicts progression to end‐stage kidney disease
Source: Clin Genet. 2022 Oct 21;103(2):214–8. doi: 10.1111/cge.14235 (PMC10092499; doi:10.1111/cge.14235)
Supplement: Supplementary file 1 — APPENDIX S1. Supporting Information [file CGE-103-214-s003.docx]

**Supplementary**

Computational Methods

All simulations were performed using the GROMACS 2021.2 (https://doi.org/10.1016/j.softx.2015.06.001) software using the Amber14SB (https://doi.org/10.1021/acs.jctc.5b00255) force field and TIP3P waters. Structures were dimerized using the GRAMM-X webserver (https://doi.org/10.1093/nar/gkl206). Protonation was performed using PROPKA 3.1 (https://doi.org/10.1021/ct200133y). The system was solvated in a cubic box using a 1.2 nm radius from the protein edge and neutralized with added ions to 0.15 M NaCl concentration. Starting structures were minimized using 5000 steps steepest descent energy minimization followed by 5000 steps conjugate gradient energy minimization. Hydrogens were constrained using the LINCS algorithm. The system was equilibrated using 100 ps NVT to 300 K using velocity-rescaling thermostat (https://doi.org/10.1063/1.2408420) followed by 100 ps NPT using the Berendsen barostat with 1 bar reference pressure. Production runs were performed in the NPT ensemble with a timestep of 2 fs for at least 600 ns per replica using Nosé-Hoover thermostat and Parinello-Rahman barostat with 1.0 ps and 2.0 ps time coupling constants respectively. Particle Mesh Ewald real-space summation cut-off was set to 1.2 nm and Lennard-Jones 12-6 interactions were cut-off at 1.2 nm with a corresponding force-switch function at 1.1 nm. Principal component analysis was performed using covar and anaeig functions from GROMACS.

Initial protein structures were taken from PDBID: 2JDW (https://doi.org/10.1093/emboj/16.12.3373) with mutant structures generated using PyMol. Distances between residue 320 on each monomer were measured between sidechain CB atoms using MDAnalysis. The distribution of distances between residue 320 on each homodimer were analysed using the Boltzmann distribution to estimate the free energy profile across the degree of freedom, using histogram bin-averaging over 100 bins for all mutants and WT. A reverse mutation of R322P back to WT (R322P_P322R) at the end of the R322P simulation, followed by an additional 600 ns simulation time, was performed for further validation (Supplementary Figure 1C). The starting structures for all variants have a starting residue 320 CB atom distance of 11.6 Å, except for the R322P_P322R reverse mutant, with a starting distance of 3.8 Å. Only partial recovery of the WT distribution was observed in the reverse mutant, with destabilization of the “close-contact” conformation but only low probability sampling of “distant” conformations, indicating the backward-transition was occurring but slowly. We expect this incomplete recovery is due to insufficient sampling, attributed to the size of the P322R mutation relative to prior mutations and limitations of conventional MD. We therefore limit our analysis to our variant simulations at the same starting configuration to approximately standardize the starting configuration bias.
