# Supplementary material for: A novel variant in GATM causes idiopathic renal Fanconi syndrome and predicts progression to end‐stage kidney disease
Source: Clin Genet. 2022 Oct 21;103(2):214–8. doi: 10.1111/cge.14235 (PMC10092499; doi:10.1111/cge.14235)
Supplement: Supplementary file 2 — FIGURE S1. Molecular dynamics results of GATM B4‐B4 dimer mutants Free‐energy values for all T336A (a), P320S (b), and R322P_P322R (c) mutants across residue 320 CB atom distances, each replica plotted separately. (def) Free‐energy values for all mutants and WT across top three principal components generated from backbone Cα atom trajectories, averaged across all three replicas [file CGE-103-214-s001.pdf]

Supplementary Figure 1 | Molecular dynamics results of GATM B4-B4 dimer mutants

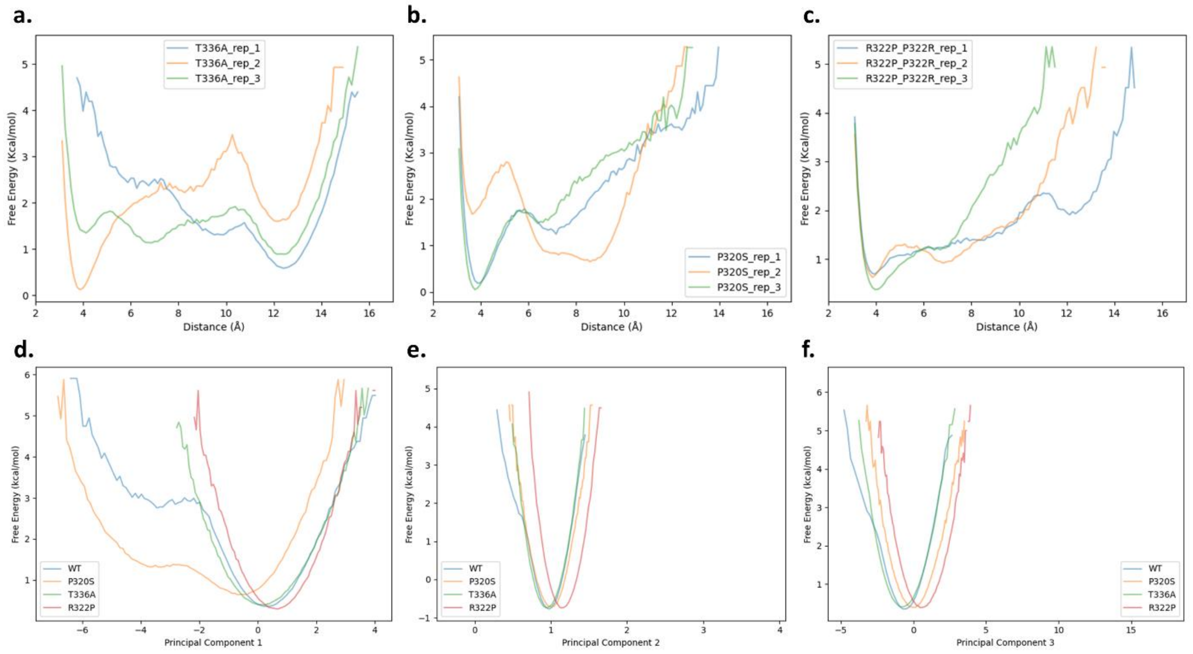

Free-energy values for all T336A (a), P320S (b), and R322P_P322R (c) mutants across residue 320 CB atom distances, each replica plotted separately. (def) Free-energy values for all mutants and WT across top three principal components generated from backbone Cα atom trajectories, averaged across all three replicas.
