# Supplementary material for: A novel variant in GATM causes idiopathic renal Fanconi syndrome and predicts progression to end‐stage kidney disease
Source: Clin Genet. 2022 Oct 21;103(2):214–8. doi: 10.1111/cge.14235 (PMC10092499; doi:10.1111/cge.14235)
Supplement: Supplementary file 3 — TABLE S1. Genetic causes of renal Fanconi syndrome [file CGE-103-214-s002.docx]

**Supplementary Table 1** | Genetic causes of renal Fanconi syndrome

| **Gene** | **OMIM** | **Disorder** | **Inheritance** | **Clinical features** | **Kidney failure** |
| --- | --- | --- | --- | --- | --- |
| *Systemic disorders with RFS* | | | | | |
| *ALDOB* | 229600 | Fructose Intolerance | AR | Vomiting, hypoglycaemia, hyperuricaemia, hepatomegaly | Kidney impairment if uncontrolled |
| *ATP7B* | 277900 | Wilson disease | AR | Cirrhosis and neurological disease, Kayser-Fleischer rings, hypercalciuria and nephrocalcinosis | No |
| *CLCN5, OCRL* | 300009, 300555 | Dent disease I, II | XLR | Hypercalciuria, hyperphosphaturia, proteinuria, and aminoaciduria, rickets | Yes |
| *CTNS* | 219800 | Cystinosis | AR | Poor growth, myopathy, corneal erosions and crystals, pancreatic insufficiency, male infertility | Yes |
| *FAH* | 276700 | Tyrosinaemia | AR | Poor growth, progressive liver disease, liver cancer, hypophosphataemic rickets | No |
| *GALT* | 230400 | Galactosaemia | AR | Liver dysfunction, jaundice, hepatosplenomegaly, encephalopathy, sepsis | No |
| *GLUT2* | 227810 | Fanconi–Bickel syndrome | AR | Failure to thrive, hepatomegaly, hypoglycaemia, rickets | No |
| *HNF4A* | 125850 | MODY1 | AD | Diabetes mellitus, mutation R76W shows RFS | No |
| *OCRL* | 309000 | Lowe syndrome | XLR | Failure to thrive, cataracts, hypotonia, developmental delay | Yes |
| *VPS33B, VIPAR* | 208085, 613404 | ARC syndrome | AR | Failure to thrive, global developmental delay, arthrogryposis, platelet abnormalities, cholestasis | Kidney dysfunction |
| *Isolated RFS* | | | | | |
| *EHHADH* | 615605 | Fanconi renotubular syndrome 3 | AD | Rickets, impaired growth, glucosuria, generalised aminoaciduria, phosphaturia, metabolic acidosis, and low molecular weight proteinuria, preserved kidney function | No |
| *SLC34A1* | 613388 | Fanconi renotubular syndrome 2 | AR | Predominantly renal phosphate wasting, severe rickets, short stature | Yes |
| *GATM* | 134600 | Fanconi renotubular syndrome 1 | AD | Proximal renal tubulopathy, fibrotic kidneys, progression to kidney failure | Yes |
| *NDUFAF6* | 618913 | Fanconi renotubular syndrome 5 | AR | Proximal renal tubulopathy, rickets, progression to kidney failure, short stature, pulmonary fibrosis | Yes |

*Table adapted from Klootwijk et al.^16^ Abbreviations: AD – autosomal dominant; AR – autosomal recessive; ARC syndrome – arthrogryposis, renal dysfunction and cholestasis syndrome; MODY1 – maturity onset diabetes of the young type I, RFS – renal Fanconi syndrome; XLR – X-linked recessive.*
